# Treatment and outcome of toxic epidermal necrolysis in 32 Chinese patients: a hospital-based study

**DOI:** 10.1186/2045-7022-4-S3-P10

**Published:** 2014-07-18

**Authors:** Ting Xiao, Li-Ming Zhang, Yan Zhao

**Affiliations:** 1 The First Affiliated Hospital of China Medical University, China; 2 The First Affiliated Hospital of China Medical University, Dermatology, China

## Background

Toxic epidermal necrolysis (TEN), the most severe drug reaction, has been reported to be associated with considerable mortality rate. In the past 15 years, the use of intravenous immunoglobulin (IVIG) have improved the prognosis of TEN.

## Method

We retrospectively studied 32 patients with TEN in a Chinese tertiary medical center between July 1, 2008 and June 30, 2013 to evaluate the clinical manifestations, laboratory tests, treatment of TEN and prognostic factors for the mortality of TEN.

## Results

Antibiotics (n=12) were the most common offending drugs, followed by non-steroidal anti-inflammatory drugs (n=8), anti-rheumatic drugs (n=2) and Chinese herbs (n=2). Hypertension (34.4%, n=11) and diabetes (21.9%, n=7) were the most common pre-existing conditions. Hepatitis B virus infection, cerebrovascular diseases and cardiovascular diseases pre-existed in 15.6% (n=5), 12.5% (n=4) and 12.5% (n=4) of the 32 patients, respectively. TEN showed higher incidence in the patients with HBV infection. All 32 patients were treated initially with systemic corticosteroids ranging from 50 mg to 150 mg daily. Sixteen patients were treated with combined IVIG (400 mg/kg/day, for 5 days). Twenty-nine patients were cured without or with different sequelae. The mortality rate was 9.38% (n=3). The application of IVIG reduced the estimated mortality of TEN to 17%. Delayed use of IVIG, elevated level of urea nitrogen and early onset of the rashes after taking offensive drugs were associated with the mortality of TEN.

## Conclusion

Systemic corticosteroids in combination with IVIG were effective for TEN in Chinese patients. TEN showed higher incidence in Chinese patients with HBV infection. Delayed use of IVIG, elevated level of urea nitrogen and early onset of the rashes after taking offensive drugs correlated with poor prognosis of TEN.

